# Autophagy Regulation and Photodynamic Therapy: Insights to Improve Outcomes of Cancer Treatment

**DOI:** 10.3389/fonc.2020.610472

**Published:** 2021-01-20

**Authors:** Waleska K. Martins, Renata Belotto, Maryana N. Silva, Daniel Grasso, Maynne D. Suriani, Tayná S. Lavor, Rosangela Itri, Mauricio S. Baptista, Tayana M. Tsubone

**Affiliations:** ^1^Laboratory of Cell and Membrane, Anhanguera University of São Paulo, São Paulo, Brazil; ^2^Perola Byington Hospital Gynecology - Lasertherapy Clinical Research Department, São Paulo, Brazil; ^3^CONICET, Instituto de Estudios de la Inmunidad Humoral (IDEHU), Universidad de Buenos Aires, Buenos Aires, Argentina; ^4^Institute of Chemistry, Federal University of Uberlândia, Uberlândia, Brazil; ^5^Institute of Physics, University of São Paulo, São Paulo, Brazil; ^6^Institute of Chemistry, University of São Paulo, São Paulo, Brazil

**Keywords:** photodynamic therapy, autophagy, cell death, cancer, clinical trials

## Abstract

Cancer is considered an age-related disease that, over the next 10 years, will become the most prevalent health problem worldwide. Although cancer therapy has remarkably improved in the last few decades, novel treatment concepts are needed to defeat this disease. Photodynamic Therapy (PDT) signalize a pathway to treat and manage several types of cancer. Over the past three decades, new light sources and photosensitizers (PS) have been developed to be applied in PDT. Nevertheless, there is a lack of knowledge to explain the main biochemical routes needed to trigger regulated cell death mechanisms, affecting, considerably, the scope of the PDT. Although autophagy modulation is being raised as an interesting strategy to be used in cancer therapy, the main aspects referring to the autophagy role over cell succumbing PDT-photoinduced damage remain elusive. Several reports emphasize cytoprotective autophagy, as an ultimate attempt of cells to cope with the photo-induced stress and to survive. Moreover, other underlying molecular mechanisms that evoke PDT-resistance of tumor cells were considered. We reviewed the paradigm about the PDT-regulated cell death mechanisms that involve autophagic impairment or boosted activation. To comprise the autophagy-targeted PDT-protocols to treat cancer, it was underlined those that alleviate or intensify PDT-resistance of tumor cells. Thereby, this review provides insights into the mechanisms by which PDT can be used to modulate autophagy and emphasizes how this field represents a promising therapeutic strategy for cancer treatment.

## Introduction

Cancer remains one of the most common causes of health problems worldwide with increasing rates in developed and under-developed/developing countries (1). The new cases and deaths numbers were estimated at 18.1 and 9.6 million, respectively, according to GLOBOCAN updates (2). Cancer more often affects aged people (50.8% of cases), but there is a worldwide concern about those >65 years in the near future (3, 4). Over the next 10 years, people will suffer more death from cancer than from other very common diseases, such as diabetes (5). It is clear, therefore, that although cancer treatment has considerably improved in the last decades, the fight against this disease is in urgent need of novel tools.

Cancer is a multifactorial disease and despite the many recently introduced chemo and immunotherapies the general clinical outcome and prognosis of cancer patients is not optimistic at all. Overall, novel therapies are less detrimental to the individual because they are specific in modulating different immune/biochemical pro-death modes (e.g. apoptosis), to get rid of tumor cells. Unfortunately, by a phenomenon known as chemo-adaptation and dormancy many human cancers (e.g. cutaneous melanoma, breast, head, and neck tumors) can downregulate specifically the pro-apoptotic mechanisms, worsening the outcome and the prognosis of cancer patients. In addition to proliferation and plasticity abilities, tumor cells considered “*stemness*” gradually give rise to chemoresistance *via* a distinct variety of mechanisms and pathways. For this reason, the modulation of different cell death pathways could help to define complementary or alternative strategies to those based on the activation of apoptosis.

Since all cells have membranes whose integrity is necessary for survival, therapeutic strategies that address specific oxidative damage in the membranes of organelles have great potential to avoid therapeutic resistance. Photodynamic Therapy (PDT) is a non-invasive and efficient strategy based on photophysical principles that may provide specific oxidative damage in organelles such as the endoplasmic reticulum, mitochondria, and lysosomes. Herein, we present our current knowledge regarding tumor resistance concerning the suppression of autophagic response, in an attempt to improve clinical outcomes. In this scenery, the photo-mediated pro-death autophagy emphasizes PDT as a promising therapy to deal with tumors that evade apoptosis. Undeniably, PDT has been applied with success to treat several types of human cancers with tolerable side effects. However, as PDT-resistance has increased due to distinct reasons (oxidative-scavenger response, autophagy activation, drug extrusion, and others), we will discuss the pitfalls and successes of its use, considering autophagy as a therapeutic target to improve tumor remission. Considering the PDT photophysics and photochemistry effects, as well as the photooxidative-mediated membrane damage, we will discuss the molecular mechanism for tumor-resistance, particularly focusing on the biological, molecular, and translational aspects of the PDT-related cancer treatments.

## Photodynamic Therapy (PDT)

Considering the difficulties and challenges in conventional cancer treatment, such as tumor resistance, new treatment concepts for both primary care and adjuvant therapy are highly necessary. PDT is a well-established medical procedure due to the selective cancer eradication (sparing normal cells), especially when tumor sites can be demarcated (6). The PDT advantages compared to the conventional cancer treatments include: (i) it does not seem to induce drug resistance, (ii) promote selective cancer destruction, preserving the surrounding normal tissues (iii) preserving the native tissue architecture and giving a decisively better recovery compared with surgery (iv) can be used with other therapies (7).

PDT is definitively less invasive compared to surgery, and more precise than chemotherapy and, finally, as opposed to radiotherapy, may be repeated several times (8). A photosensitizer (PS) molecule can be administered intravenously, intraperitoneally, or topically to the patient, and the tumors tissue sites are selectively irradiated. Although these components (i.e., PS and light) are harmless alone, when combined they provide localized antitumor therapy. This avoids damage to healthy cells thus preventing side effects. The combination of PS and light results in the generation of reactive excited states (singlet and triplet excited states) as well as several reactive oxygen species (ROS), such as singlet oxygen ${(}^{1}O_{2})$, hydroxyl radical ${(}^{\cdot }OH)$, superoxide ion $\left(O_{2}^{-\cdot }\right)$, and hydrogen peroxide (H_2_O_2_). These reactive species can efficiently oxidize and irreversibly damage targeted tumor tissues/cells (9–11).

**Figure 1:**
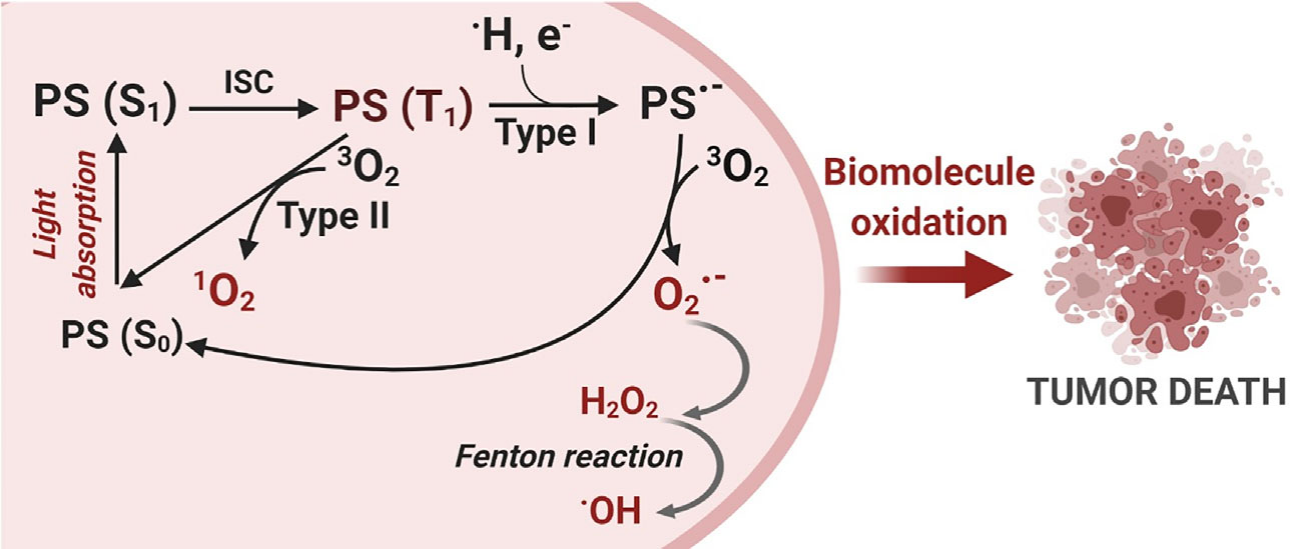
Photodynamic Therapy Mechanism. The photosensitization process starts with a photon absorption that converts the photosensitizer PS (S_0_) ground state to a more energetic state known as a singlet excited state PS (S_1_). Then, an intersystem crossing conversion (ISC) changes the PS multiplicity to a triplet excited state PS (T_1_). PS (T_1_) can interact with molecules nearby and react *via* two distinct mechanisms: Type I – electron transfer and Type II – energy transfer, generating reactive oxygen species (ROS). Finally, oxidative species damage biomolecules and can trigger cell death. Created with BioRender.com.

Light with a specific wavelength PS triggers the photooxidative process, as summarized in **Figure 1**. PS excitation through photon absorption transforms the ground state PS (S_0_) into an excited state - singlet excited state PS (S_1_). Next, PS (S_1_) can be converted into a triplet excited state (PS (T_1_), by the change in the spin of electron *via* a process known as intersystem crossing (ISC). Due to its new spin configuration, PS (T_1_) can live long enough to interact with species nearby, resulting in two main photosensitization mechanisms: (a) energy transfer to oxygen (Type II process) or (b) a directed reaction with biological substrates (Type I process). On the Type II process, energy transfer to molecular oxygen ${(}^{3}O_{2})$ yields the highly reactive oxygen state known as singlet oxygen ${(}^{1}O_{2})$, an electrophilic molecule that is often considered the main PDT performance species (10–12). Type I processes are based on reactions between PS (T_1_) and nearby biomolecules, forming a variety of products, which can start a radical chain reaction. The free radicals generated during the Type I mechanism can still react with oxygen, resulting in the production of ROS such as ·OH, $\left(O_{2}^{-\cdot }\right)$, and H_2_O_2_ (10–12).

These two reaction mechanisms, Type I and Type II, invariably involve oxygen as either a primary or a secondary intermediate reactant and are also called photosensitized oxidation reactions (11, 13). Both mechanisms may occur simultaneously, and a balance between them is important for ROS production and, in turn, determines the overall photo-cytotoxicity effectiveness of the PDT reaction (11, 14). The dominant mechanism will depend on the PS itself, the type of substrate, the distance between the PS and the oxidative targets as well as the oxygen concentration.

The PDT efficiency depends on the illumination conditions, the chemical properties, and the intra-tumoral localization of the PSs localization. Selecting a suitable device for the tumor region irradiation is a fundamental factor in PDT protocols. The main types of light sources used in PDT include lasers, light-emitting diodes (LEDs), and lamps. Each category source presents advantages and disadvantages. For this reason, a choice of proper light source needs to be carefully evaluated according to the PS, tumor location, and the light dose to be delivered (15). The geometry of the tumor area which, sometimes, is not easy to access, determines the decision of the correct light apparatus to be used (15). As an example of selecting a suitable light device, Davanzo et al. demonstrated that it is possible to obtain different PDT outcomes depending on the light source used (16). Under the same light dose exposure, they reported that continuous laser was a better light source compared to other devices (pulsed laser and LED) under the same light dose exposure (16). Indeed, other factors impact the final PDT outcome, including the amount and the type of reactive species, which is highly dependent on the photochemical and photophysical properties of the PS.

Several classes of PSs have been commonly employed in PDT, including porphyrins, chlorins, phthalocyanines, and phenothiazines (10). Each one of them presents distinct advantages and disadvantages regarding the chromophore type. For example, Photofrin™ (porfimer sodium), which is an oligomer and was the first PS approved by the FDA for the treatment of bladder cancer in Canada in 1993 (17). Its structure is not well defined, but its aqueous suspension can be applied intravenously. However, the absorption in the low ‘therapeutic window’ (between 600-800 nm) and a prolonged (~ 4 weeks) skin photosensitivity is an important side effect (17). On the other hand, phthalocyanines have a high molar absorption coefficient in the red spectral region but are not water-soluble. To deal with this limitation, a liposomal zinc phthalocyanine was developed and has been tested in phase 1 or 2 clinical trials for solid tumors (17). However, it did not reach the clinical practice, probably due to issues concerning the stability and the difficulty of large-scale liposome (18).

Since several ROS species have high reactivity, short lifetimes, and consequently small diffusion pathways (12), only those PSs nearby to the biological substrates can cause tumor photodamage (12, 19). ^1^O_2_ lifetime in pure water is ~4 µs (20), which provides a mean diffusion distance traveled by ^1^O_2_ molecules in water of less than 200 nm, without considering any other reaction with biomolecules besides its intrinsic decay. Although the average dimensions of mammalian cells are around 10-30 µm diameter, the ^1^O_2_ mediated oxidative-damage would reach only short distances, reaching specifically a target PDT organelle (12, 21). Therefore, generating amounts of ROS does not mean PS effectiveness (22). If PS is near to an intracellular target, the photo-generated ROS would oxidize biomolecules in a more specific way (22). Thus, the oxidative reactions primarily affect only nearby PS-targeted organelles (12). Also, the relative oxygen concentration may favor or disfavor ^1^O_2_ formation, which may amplify (or not) the biomolecule’s oxidation reactions (23, 24). Another parameter that drives PDT efficiency is the molecular structure of the PS. Of note, PSs belonging to the same class may have distinct properties, given the diversity of the side-groups that can be attached to the lead chromophore (25–31).

Knowing that biological membranes guarantee cell homeostasis due to their crucial role in compartmentalizing intracellular content and organelles, they are particularly important targets for PDT. The basic structural membrane elements are the lipid bilayer and the integral or linked proteins (32, 33). Both the lipids and the integral proteins display amphiphilic characteristics, explaining why PSs that exhibit amphipathic character will interact with the membrane, independent of whether it is a plasma, mitochondrial, lysosomal, or endoplasmic reticulum membrane. As an example, Engelman et al. compared porphyrins with two charged groups around the ring at position *cis* and *trans* and observed that *cis*-isomer presented a much larger binding to the membrane than predicted by water/octanol partition (log P_OW_) (34). This is because the cis-isomer has an optimized amphiphilic structure that matches the amphiphilic structures of the lipids in the bilayer. As a result, an enhanced photodynamic efficiency was perceived regardless of the type of the membrane (i.e., liposomes, mitochondria, and erythrocytes membrane) (34). Tsubone et al. also studied a series of amphiphilic PSs displaying opposite charges (negative or positive) and noticed that hydrophobic and dipolar interactions play crucial roles in defining the affinity of these molecules to membranes (12, 35). Although the increase in the alkyl chain length above certain limits leads to aggregation and decreases in the PS photoactivity, increasing the hydrophobicity up to certain limits has also been associated with enhanced cell photokilling efficiency (36–38). Another parameter that favors the PS binding in the lipid membranes is the molecule asymmetry. In Porphyrin, a peripherical group at *meta*-position was found to be more phototoxic than its *para*-isomer, mainly because the *meta*-isomer asymmetry favors the PS-membrane interaction compared to the symmetric *para-isomer* (39).

Because proteins are the most abundant biomolecules in cells, they probably act as major targets for photo-oxidation (40, 41). The main forces that govern PS-protein interaction are well described in the literature (42, 43). Phototoxic outcomes seem to depend on PS-protein interaction. Towards this end, Cozzolino et al. bound curcumin to bovine serum albumin and showed that the conjugate displays a better photodynamic effect when compared to the unbound curcumin (44). Proteins can also be used as PS carriers. Recently, it was reported a macromolecular approach of a synergistic combination of Ru-complexes on a protein carrier with subcellular mitochondria targeting groups, allows enhanced phototoxicity and efficacy (45).

Linking the PS to a monoclonal antibody allows the photodamage to be addressed to key specific molecular markers, present, for example in tumor surface. In this context, the epidermal growth factor receptor (EGFR) is a promising target for PS-immunoconjugates, considering it is commonly overexpressed in cancer cells (46). Indeed, it has been recently shown that the verteporfin-immunoconjugate (monoclonal antibody targeting EGFR) causes significantly higher levels of cell death in ovarian metastatic cancer cells (overexpressing the EGFR receptor) compared to the cell death without EGFR overexpression (47, 48). Besides the cell-surface EGFR receptors that have antibodies, such as cetuximab (49) or panitumumab (50), recent reports pointed out as promising targets in preclinical models (51) the photobiomodulation of tumor-associated regulatory T cells (52, 53).

Cellular compartments vary substantially and the photosensitizer structures determine the subcellular location of the photodamage and control cell death efficiency (12). Therefore, understanding the cellular and molecular photodynamic mechanisms can lead to an optimization in the PDT efficacy. As long as each PS has a distinctive subcellular localization profile, the PDT-mediated cell death can be modulated regarding specific oxidative stress in the targeted organelle (47). For instance, whereas CisDiMPyP incorporates into mitochondria, TPPS_2a_ accumulates mainly within lysosomes (35). Other PSs can evoke mitochondrial, lysosomal, and/or ER photodamage (35, 54–62). Such a possibility of PSs selectively inducing damage in targeted organelles is key to potentiate the photo-induced cell death (63, 64).

## Molecular Mechanisms for Tumor Resistance to PDT

**Figure 2:**
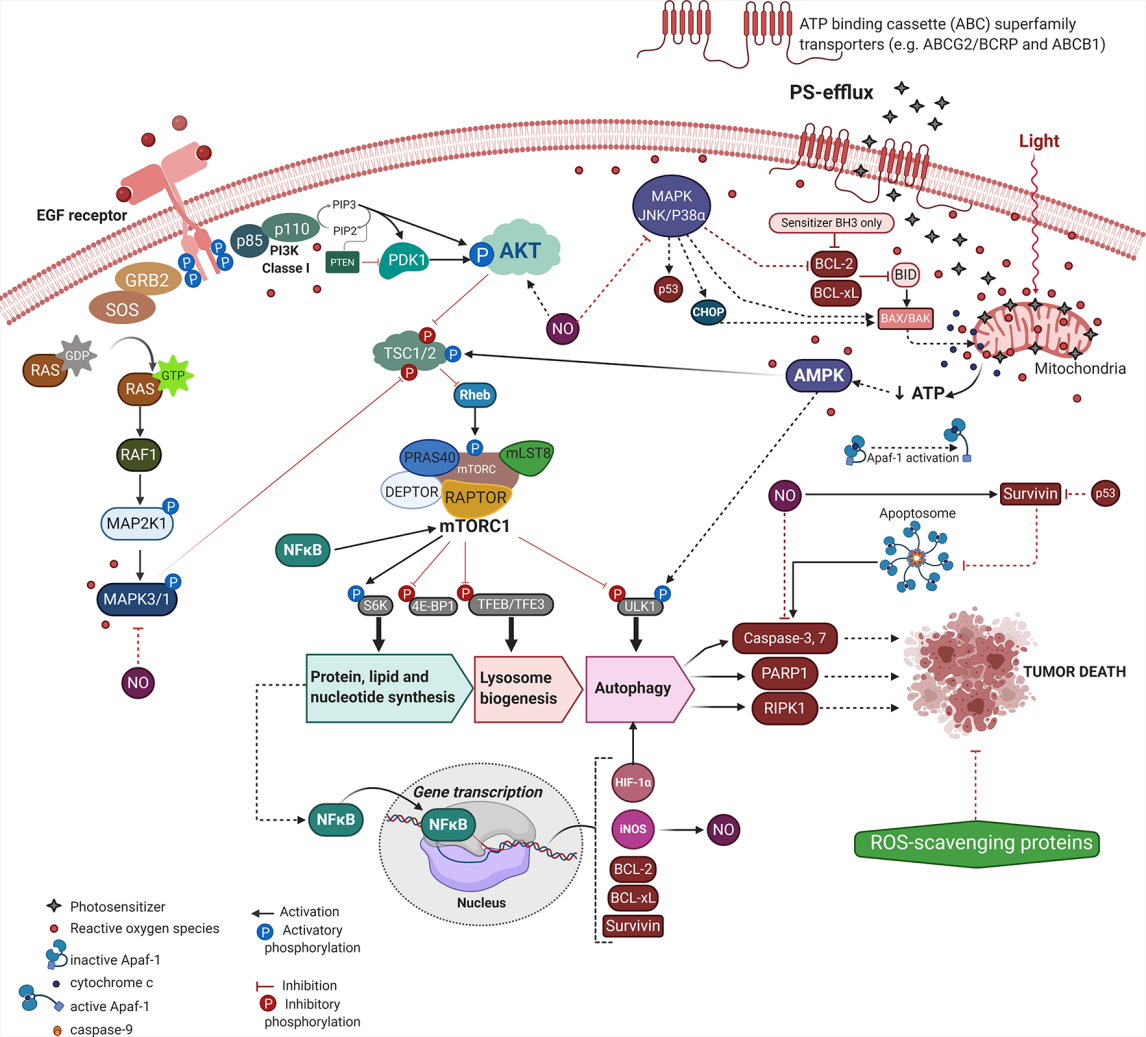
Molecular mechanisms underlying PDT-resistance of tumor cells. Created with BioRender.com.

The resistance to regulated cell death mechanisms (RCD) is one of the most prominent cancer hallmarks, intrinsically contributing to tumor recurrence and metastasis. Accordingly, the tumor relapse to current conventional chemotherapies has increased up to 2500-fold (65). The PDT approach (i.e., light energy and PS concentration) might eliminate most of the tumor cells, however, some of them may elicit their survival and dormancy, leading to phototherapeutic cancer resistance. Thus, despite PDT potentially circumventing cancer recurrence to some chemotherapies (e.g. cisplatin, dacarbazine, or 5-Fluoracil) (66–68), its promisor tumor dealing potential might also fail (69–72). Therefore, just as with other approaches similar to chemotherapy and radiotherapy, post-PDT treatment tumors are prone to become resistant and more aggressive (73, 74). Most of these mechanisms that elicit tumor PDT-resistance relies on the number of phototherapy sessions, the cell type, delivery system, and photo-physical aspects of the PS (65, 74–76). Although the PDT resistance mechanism remains elusive, we briefly consider the main molecular mechanisms underlying the tumor defense against the photooxidative damage and PS uptake (**Figure 2**). We also pointed out the new PDT approaches to deal with tumor recurrence and maximize the phototherapeutic efficacy.

Tumoral tissues might acquire an intrinsic resistance to treatment or activate alternative compensatory signaling pathways to handle cytotoxicity (73). Indeed, most of those resistant mechanisms comprise an adaptative response to the therapeutic-mediated extrinsic stresses, including mutations, altered genetic and epigenetic profiles, dysregulation of regulatory proteins of apoptosis or autophagy, dormancy, surrogation of the chemotherapeutic targets, drug efflux capacity, and stimulation of compensatory signaling or mediated repair pathways (76–80). Moreover, we can focus on tumor relapse related to the tumoral microenvironment, limited incorporation of the PS, hypoxia, and low penetration of radiation into tumoral mass (73).

In many cases, cell morphology, cytoskeleton, and cell adhesion changes have been observed in cells or tissue under photosensitization, which was correlated to significant impairment of migratory and invasive behaviors (81). The photo-mediated alterations into cytoskeleton (e.g. shorter stress fibers, decreased number of dorsal fibers, loss of cell-to-cell interactions, and epithelial morphology) ascribes less invasiveness and migratory properties to tumor cells, which lead to higher cellular plasticity and PDT resilience (81). Paradoxically, cytoskeleton alteration associated with invasion and metastasis might result in PDT-resistance (81). Such discrepancies can occur due to higher tumor heterogeneity, as well as the presence of hypoxic regions that may impair the PDT-overcome in innumerous ways beyond the limitation of oxygen, one of the components of phototherapy (**Figure 1**).

In an elegant model using heterotypic spheroids composed of human colorectal SW480 cancer cells and fibroblast, Lamberti et al. demonstrated that the tumor-stroma interaction with a hypoxic environment significantly impairs the 5-ALA metabolism, and so reduces the production of the endogenous PpIX (Protoporphyrin IX), the photosensitizer molecule (82). In this context of oxygen deprivation, HIF-1α is the key player and, despite conferring adaptability to hypoxia, it might also assign resistance to PDT by at least avoiding intracellular PS accumulation. Additionally, the HIF-1α mediated resistance could be induced by the PDT itself (83). In colorectal cancer cell spheroids, the PpIX-PDT can activate the MAPK1/ERK2 and MAPK3/ERK1 pathway as an adaptative and survival mode to resist the mitochondrial photooxidative damage. This molecular event results in the transcriptional activation of HIF-1α, suggesting that the ROS-MAPK1/3-HIF-1α axis may be a solution for PDT-resistance (83). It is worth noting that autophagy induction in response to PDT might be also related to HIF-1α. The simple HIF-1α stabilization induces autophagy in colon Caco-2 and SW480 cancer cells and significantly increases cell survival following PpIX-PDT (84). The autophagy activity is dependent on HIF-1α since this transcription factor recognizes a hypoxia response element (HRE) in the promoter of expression of the vacuole membrane protein 1 (VMP1), a protein capable of inducing the formation of autophagosomes (84) (**Figure 2**).

The cancer expression profile of drug-efflux mediators has been involved in multidrug tumor-resistance (MDR) against chemotherapeutics such as imatinib, doxorubicin, and mitoxantrone, as well as PDT (79, 85–91). The ATP binding cassette (ABC) superfamily transporters (e.g. ABCG2 and ABCB1) were found to extrude PS out of the tumor cells (86–88). Despite some mutations on ABCG2 (e.g. R482G, R482T) not affecting PS transport, the Q141K polymorphism may explain increases in the patient photosensitivity to PDT on account of a lower PS efflux (89, 90). On the other hand, the ABCG2 overexpression has been suggested to render the incorporation of some photosensitizers with chemical similarity to pheophorbide A (PhA), including Ce6, MPPa, and 5-ALA (89). Noteworthy, the photosensitizers m-THPP and m-THPC may provide a more effective PDT response even in ABCG2-overexpressing bronchoalveolar carcinoma H1650 MX50 cell line (89). To improve PDT-efficacy considering the ABCB1-mediated PS extrusion, there are several new protocols in development including those on zinc phthalocyanine tetrasulfonic acid and nanotechnology approaches (79, 92). Another way to overcome PS-efflux is to promote a multifunctional drug delivery system (e.g. endocytosis), in which lysosome highlights as a targeted organelle (69), as well as the PDT combination with ABCG2 inhibitor Ko143 (93). Human glioblastoma T98G cells with the highest *ABCG2* expression levels showed relevant synergic death after the PpIX-PDT plus Ko143 in response to increased 5-ALA incorporation (93).

Recently, TPPS-loaded nanogels through its endocytic internalization and pH-sensitive framework might elude photo-oxidation toward multidrug-resistant cancer cells (94). Noteworthy, this approach also remarkably modulates autophagy, whose inhibition may alleviate PFKFB3-elicited tumor dormancy (95). PFKFB3 functions as a regulator of cyclin-dependent kinase 1, linking glucose metabolism to cell proliferation and survival, as well as apoptosis prevention. Depending on the physicochemical PS properties (e.g. pKa), the endo/lysosomal entrapment phenomena may occur during PDT (86), as reported by multiple hydrophobic weak-base drugs (e.g. sunitinib, doxorubicin) (96, 97).

Several strategies have been proposed to overcome PDT-resistance on PS-specificity (70–72, 79). Most of them adjust the PS chemical structure by targeting specific membrane components, which may lessen PS-extrusion. For example, the covalent introduction of a phospholipid to generate porphyrin-lipid derivatives might deal with ABCB1-mediated BPD extrusion and alleviate PDT-resistance of tumor cells (88). It is noteworthy that the ‘unconjugated’ form of the same porphyrin-lipid does not mitigate the BPD efflux by ABCB1 in breast cancer cells (88). The covalent conjugation of indocyanine green (ICG) and TNYL peptide onto the surface of gold nanospheres (HAuNS) was found to overcome the PDT-resistance (72). Also, Liu et al. have proposed the molecular linkage of the nano photosensitizer to a BCL-2 inhibitor as an adjuvant intervention strategy to increase the PDT efficacy in relapsed-tumor cells (71). Kralova et al. demonstrated that PDT-resistance may be related to PS lipophilicity (86). While glycol porphyrins suffer ABCB1-mediated drug-extrusion, the elicited PDT-resistance associated with the highest lipophilic structure of termoporfin relies on the PS-lysosome sequestration (86).

Also, the protein dysregulation involved in PS-influx must be considered. The 5-ALA influx transporters, such as ABCB6 and SLC15A1/PEPT1, play a pivotal role in the PS-uptake, whose overexpression might increase the PDT efficacy depending on the PS type and the subcellular specificity (91, 98). Thereby, their genetic profiles might determine phototherapy efficacy in dormant cancer cells responsible for disease latency, late metastasis, and tumor relative relapse to chemotherapy and radiation (80, 99).

Aside from less PS accumulation or reduction on ROS generation, the tumor molecular adaptation regarding signaling pathways (e.g. MAPK/JNK/p38α, AMPK, and AKT/mTOR) have also provided PDT-resistance with crosstalk between apoptotic machinery (e.g. BCL-2, BCL-xL, survivin, caspases, and PARP1) and autophagy, as summarized in **Figure 2** (60, 74, 76, 100–103). To overcome ATP depletion due to mitochondrial photo-oxidation, tumor cells activate the canonical energy-sensing AMPK mechanism (104). After phosphorylation AMPK becomes active and leads to Rheb/mTORC1 inhibition with consequent induction of lysosome biogenesis and autophagy, which may dictate the tumor PDT-resilience (102, 104). Also, the acquired tumor resistance to TPCS_2a_-PDT likely occurs due to higher expression of the EGF receptor (i.e., EGFR) and loss of the MAPK/p38 inducing death pathway (76). Indeed, by targeting EGFR the TPCS_2a_-PDT-resistance is significantly reduced regardless of the tumor adaptation respecting the cell death mechanism (e.g. apoptosis, necroptosis, or autophagy), **Figure 2** (76).

The phototoxic PDT-effects might be abrogated by antioxidant defense mechanisms, including ROS-scavenger proteins glutathione, ferrochelatase (FECH), heme oxygenase (HO-1), glutathione peroxidase 4 (GPX4), and glutathione S-transferase Pi 1 (GSTP1) (93, 105). Besides, the heat shock protein 27 (HSP27) may also play a pivotal role in tumor resistance to the mediated-photooxidative stress, e.g. against Photofrin™ (106) or 5-ALAm-PDT, which was related to the activation of autophagy-based recurrence (107). Paradoxically, its downregulation leads to a relevant decrease in HSP70 expression under hematoporphyrin-PDT, which was associated with an increase in autophagy (108) or apoptosis lessening (109).

Another type of resistance involves nitric oxide (NO) generation through inducible nitric oxide synthase (iNOS/NOS2) in tumor cells. NO has a short life (i.e., <2 s in H_2_O) depending on the ^3^O_2_ concentration, and is freely diffused as a bioactive free radical interacting with other biomolecules and membranes by hydrophobic partitioning. Besides, NO reversibly impairs mitochondrial respiration through competitive cytochrome oxidase inhibition (110). Several reports revealed that the photooxidative stress may activate the inducible NO synthase isoform (i.e., iNOS), which catalyzes the L-arginine conversion to citrulline and NO in a Ca^+2^-independent manner and the expense of NADPH and O_2_ (111). The PDT-induced iNOS activation may virtually increase NO at the micromolar concentration, which reacts with superoxide ion $\left(O_{2}^{-\cdot }\right)$ to give peroxynitrite (ONOO^−^), a strong oxidant that damages both DNA and unsaturated membrane phospholipids, as reviewed by Tsubone et al. (112). Such NO has been shown to modulate tumor PDT-resistance, which was first demonstrated for Photofrin™ through an *in vivo* preclinical test to treat cancer (113). Subsequently, several reports revealed that the iNOS-derived NO might play a pivotal role in the adaptation and survival of breast (100, 114–117), glioma (103, 118, 119), and prostate (120) cancer cells to 5-ALA-PDT oxidative stress. Autophagy activation may modulate the iNOS expression in response to the suppression of AKT/mTOR signaling *via* ROS generation by UCNPs/Ce6-PDT (121).

5-ALA-PDT also triggers NO-adaptative resistance *via* activation of the PI3K/AKT signaling, leading to NFκB-mediated transcription of iNOS, **Figure 2** (100, 120, 122). Such iNOS upregulation increases NO that modulates cytoprotection against the photo-stress, including apoptosis abrogation, MAPK1/3 deactivation, invasion/migration, and tumor pro-growth (100, 103, 114–120). The adaptative response of tumor cells to PDT-generated oxidative stress (i.e., increased NO) correlates with inhibition of the pro-apoptotic role of MAPK/JNK/p38α pathway (100, 114), with consequent downregulation of the anti-apoptotic proteins survivin, BCL-2, and BCL-xL, lessening the caspase-dependent apoptosis (117, 122). As proposed by *Girotti* the tumor antagonism mediated by the iNOS/NO axis may promote further PDT-resistance pro-growth, invasion, and migration of tumor cells, leading to cancer recurrence (123). To improve 5-ALA-PDT outcome several approaches have been proposed, including iNOS non-specific activity inhibitors (e.g. L-NAME or L-NNA), iNOS specific inhibitors (e.g. 1400W or GW274150), NO scavenger (e.g. cPTIO), NFκB inhibitor (e.g. Bay11) or iNOS-knockdown (100, 103, 114–120).

Another molecular mechanism related to tumor adaptation and resistance against 5-ALA-PDT photo-oxidation relies on the anti-necrotic role of NFκB *via* the increase in AKT/mTOR signaling, at least for glioblastoma U87 and LN18 cells (103). Likewise, 5-ALA-me through photooxidative stress enhances HIF-1α that alleviates cell demise due to an increase in expression of VMP1, which plays a vital role in autophagy initiation (84, 124). On the other hand, 5-ALA-photoinduced stress may activate autophagy *via* AMPK signaling, whose chemical negative regulation results in less caspase-9 activity, and in turn, death suppression, and PDT-resistance (104). Upon ER photoinduced stress autophagy activation contributes to adaptation and rescue of the cellular homeostasis upon RO damage by hypericin-PDT (60). Autophagy abrogation in ATG5-silenced cells increases the PERK/eIF2α/CHOP cascade in response to augmentation of chaperones HSPA5 or GRP78/BiP after hypericin-PDT induced ER-stress (125). It seems that the correlation between the levels of proteotoxicity and the amount of ROS relies on selective autophagy towards the damaged endoplasmic reticulum (i.e., reticulophagy). Therefore, any process that alleviates proteotoxicity and ER-stress might lead to functional consequences, including anticancer immunity (125–127), as well as chemosensitivity (128). For more detailed information see our recent review (112).

Whereas autophagy suppression (i.e., *ATG5* knockdown or 3-MA) increases tumor cells’ death, in non-malignant cells (e.g. fibroblasts and murine embryonic fibroblasts, MEFs) autophagy-deficiency paradoxically alleviates mitochondrial cytochrome *c* release, caspase 3 activation, PARP1 cleavage, and turn apoptosis induction. Besides, such ATG5-deficiency leads to clearance of oxidized proteins and reduces photokilling by hypericin-PDT probably through up-regulation of LAMP2A, a receptor for another type of autophagy, i.e., chaperone-mediated autophagy (60). Nevertheless, LAMP2A knockout implicates in apoptotic death correlated with the upregulation of caspase-3 and poly(ADP-ribose) polymerase 1 (PARP1) (60). There is interesting crosstalk between autophagy regulation and PARP1 that may evoke resistance to PDT (101). Besides, cancer stem cells (CSC) are essential players for PDT-resistance and tumor regeneration. Consequently, Wei et al. demonstrated that autophagy in colorectal cancer stem-like cells promotes resistance to PDT-induced apoptosis (129). By isolating PROM1/CD133^+^ stem-cells they revealed a significant and specific increase in autophagy in response to PpIX-PDT (129). Interestingly, autophagy inhibition and PDT concomitantly elicit higher apoptosis induction, and so, *in vivo* tumorigenicity alleviation (129). Therefore, autophagy plays dichotomic roles in the determination of the cellular resistance or sensitization to PDT-mediated oxidative-stress. This paradigm will further be considered along with the studies discussed in the next section.

## PDT-Mediated Autophagy Regulation in Tumor Cells

**Figure 3:**
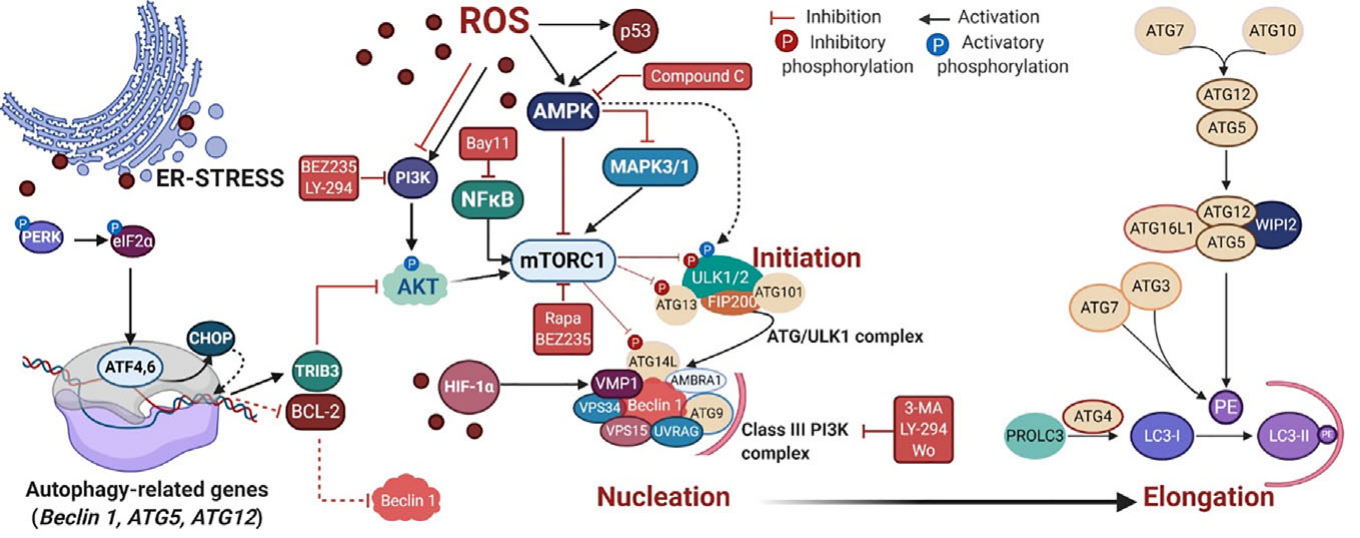
PDT-mediated autophagy regulation in mammalian cells. The reactive oxygen species (ROS)-mediated mechanism covers the autophagy regulation *via* several signaling cascades, including the energy-sensing AMPK and the PI3K/AKT/mTOR pathways. Following mTOR inhibition, the autophagy initiation starts through the activation of the ATG/ULK1 complex that translocates to the ER membrane and recruits ATG9, providing membrane components to the phagophore (130, 131). ULK1 complex can also be alternatively activated by AMPK (132, 133). In the nucleation process, following activation of the Class III PI3K complex I, occurs the production of phospholipid phosphatidylinositol3-phosphate (PI_3_P) which recruits PI_3_P-binding proteins (e.g. WIPI2), resulting in the change of the ER membrane structure with its elongation to form a phagophore (134). The elongation step relies on the generation of the soluble cytosolic LC3-I that becomes LC3-II after conjugation to the head group of the lipid phosphatidylethanolamine (PE), which occurs through a cascade of ubiquitin-like reactions involving ATG enzymes (e.g. ATG7, ATG3, and ATG5-ATG12-ATG16L). Next, LC3-II is attached to the lumenal and cytosolic surfaces of autophagosomes. The drug mediators that activate (BEZ235, rapa, and LY-294) or inhibit (3-MA, compound C and wo) autophagy machinery are depicted in red. Rapa = rapamycin, LY-294 = LY-294002, wo = wortmannin. Picture created with BioRender.com.

PDT triggers autophagy (or macroautophagy) in tumor cells by suppressing the AKT-mTOR signaling (60) or up-regulating the AMPK pathway (102, 104), as summarized in **Figure 3**. The negative effects of the TSC1/2 complex on the mTORC1 activator Rhe may be regulated by AMPK or AKT signaling (**Figure 2**). Also, autophagic machinery may be transcriptionally regulated (e.g. ATF4, ATF6, CHOP, and p53) in response to cytoplasmic or organelle photo-oxidation (128, 135, 136). Photo-oxidation enhances HIF-1α/VIMP1-mediated autophagy induction (84, 124). There are other pathways triggered by PDT capable of regulating autophagy machinery, e.g. NFκB (103) and MAPK1/3 (74) (**Figure 3**).

Depending on the extent of mitochondrial photodamage, tumor cells elicit mitophagy to rescue cellular homeostasis through clearance of oxidized or depolarized mitochondria. Mitophagy has several distinct variants (*i.e.*, type 1, 2, and 3) and prevents the release of proapoptotic proteins, generation of toxic mitochondrial-derived ROS, and futile ATP hydrolysis (137–140). A primary cellular response following the mitochondrial photodamage is the recruitment of the E3 ubiquitin ligase PRKN*/*parkin to the mitochondrial outer membrane, which depends on PINK1 (59, 141). Once recruited, PRKN ubiquitinates several outer membrane proteins marking mitochondria for 2 mitophagy (140, 142).

The cellular responses against the photo-stress also involve selective autophagy known as reticulophagy that removes oxidized ER subdomains. Despite not yet being fully understood, there are two main ER-resident proteins prone to interact with LC3-II, i.e., reticulophagy regulator 1 (RETREG1/FAM134B) and cell cycle progression 1 (CCPG1) (143). During the PDT-mediated reticulophagy, ATF4 or CHOP upregulates the expression of TRIB3 or autophagy-relevant proteins (ATG5, ATG12, and Beclin 1), as well as downregulates the expression of BCL-2 (62, 135, 136). The PDT-mediated autophagy can be chemically regulated by some drugs as depicted in **Figure 3**, such as rapamycin, BEZ235, LY-294000, Compound C, 3-MA, and wortmannin (56, 60, 100, 103, 104, 144–147).

PDT of early response genes by the hyperactivation of the survival pathway, resulting in overexpression of anti-apoptotic (BCL-2, survivin, BCL-xL) or autophagy-related proteins, evoking PDT-resistance (68, 122). Recently, the tumor resistance to several antitumor agents (e.g. cisplatin, oxaliplatin, carboplatin, doxorubicin, etoposide, rapamycin, everolimus, alpelisib, pictilisib, and AZD8055) was related to elevated and sustained activation of the PI3K/mTOR signaling pathway (148). Curiously, in these PI3K/mTOR-activated cells, the promotion of energy metabolism stress (e.g. 2-DG/DCA) led to apoptosis due to the sustained blockage of the pro-survival autophagy (148). Hence, photo-damaging organelles, such as mitochondria, lysosomes, or reticulum endoplasmic seem to be amenable to mediate death in drug-unresponsive tumors.

Evidence relating to the important role of autophagy in the PDT context continues to accrue (63). Owing to the high reactivity of photogenerated ROS, selective autophagy is initiated to remove oxidatively damaged organelles, such as mitochondria (i.e., mitophagy), lysosomes (i.e., lysophagy), endoplasmic reticulum (i.e., reticulophagy), and, or peroxisomes (i.e., pexophagy), which are intracellular targets of several photosensitizers (149). Despite controversial findings concerning autophagy activation *via* ROS generation following PDT, there is now a consensus about the underlying mechanisms regarding cytoprotection or death.

**Figure 4:**
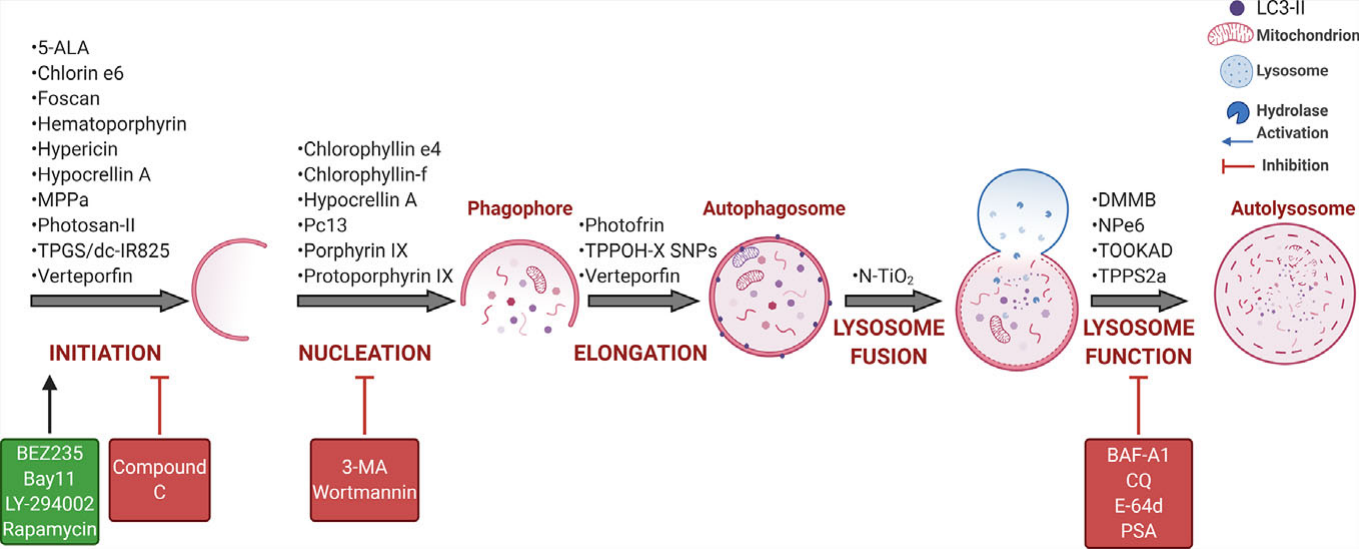
PDT-mediated autophagy regulation in tumor cells. Several PSs were described as phototherapeutic modulators of autophagy flux, which may be further activated (green) or inhibited (red) by some drugs, including the Class I PI3K/mTOR inhibitor (BEZ235), NFκB inhibitor (bay11), pan-the Class I PI3K inhibitor (LY-294002), or mTORC1 inhibitor (rapamycin), AMPK inhibitor (compound C), Class III PI3K/VPS34 inhibitor (3-MA or wortmannin) and lysosome inhibitor (BAF-A1, CQ, E-64d, or PSA) (104, 150–156). BAF-A1 also blocks the lysosome fusion with autophagosomes that occurs independently on intralysosomal pH and relates to reduced ATP2A/SERCA activity (152, 157). For more details, see **Tables 1** and **2**. Art was created with BioRender.com.

Over the past ten years, some questions have been addressed by several authors using cancer cell lines and distinct PDT protocols. As summarized in **Figure 4**, PDT protocols with different PSs were found to therapeutically modulate autophagy. Note also that the complexity of autophagy and numerous steps allows for several possibilities of intervention, but the performance in PDT does not come to simple conclusions, i.e., a better understanding of its role is still necessary. To avoid controversial analysis of the real role of autophagy (i.e., cytoprotective *versus* death routine), the autophagy community appeals to a straightforward effort in following robust guidelines to monitor autophagy (158–160). As summarized in **Tables 1** and **2**, numerous *in vitro* or *in vivo* studies have been conducted to describe the autophagic pivotal role in PDT. Herein, we will briefly discuss this paradigm.

**Table 1:** PDT-mediated autophagy: cytoprotective role and modulatory guidelines to increase tumor outcome.

PS	Target	Experimental model	Autophagy activation	Therapeutic modulation of the autophagy machinery		Outcome	Ref.
				Chemical	Genetic		
5-ALA	Mito	Human glioblastoma (U87 and LN18) and breast cancer (COH-BR1)	PI3K/AKT/mTOR	BAF-A1 Wortmannin Bay11	ATG7^A^	↑apoptosis, ↑necrosis ↑LDH	(100, 103)
Chlorophyllin e4	Lyso	Human bladder cancer (5637 and T24)	Beclin/LC3-II increase	3-MA BAF-A1		↑apoptosis	(55)
Chlorophyllin f	Lyso Mito	Human bladder cancer (5637 and T24)	LC3-II increase	3-MA		↑apoptosis	(54)
Hematoporphyrin	Memb.	Human oral cancer (Fadu)	AKT/mTOR	3-MA		No effect	(101)
Hypericin	ER	Human cervix carcinoma (HeLa)	AKT/mTOR	3-MA	ATG5^A^	↑apoptosis ↑cleaved PARP1 ↑caspase 3	(60)
Hypericin low dose (<100 nM)	ER	Human oxaliplatin-resistant colon cancer (HCT116, HCT8) and HCT116/L-OHP murine model	↑GRP78/↓LC3II/I ↓SQSTM1	3-MA 4-PBA	Beclin 1^A^ CHOP^A^	↑apoptosis ↓out-growth ↓Ki67 ↑oxaliplatin sensitization	(128)
Hypocrellin A	Mito	Human cutaneous squamous carcinoma (cSCC)	JNK/NFκB pathway	3-MA Bay11		↑apoptosis ↓BCL2 ↑caspase 3 ↑BAX	(146)
Pc13	Mito	Human melanoma (A375)	BCL-2/LC3-II increases	CQ 3-MA Wortmannin		↑apoptosis ↑cleaved PARP1	(74)
Photofrin™	Mito	Human cervix carcinoma (HeLa) and breast cancer (MCF7)	LC3-II increase	3-MA BAF-A1	ATG5^B^ ATG5^C^	↑apoptosis ↑cleaved PARP1 ↑caspase-3	(161, 162)
Photosan-II	Mito	Human colorectal cancer (HCT116 and SW620), and SW620 derived xenografts	ATK/mTOR and AMPK pathway	CQ	ATG7^A^	↑apoptosis ↑cleaved PARP1 ↑LC3-II ↓Tumor mass	(102)
Porphyrin IX	Mito	Human colon cancer (HCT116)	BCL-2/Beclin 1/ATG7/LC3-II	CQ	ATG7^A^	↑apoptosis ↑caspase 3 ↑LC3-II ↑SQSTM1	(163)
Protoporphyrin IX	Mito	Human colon cancer (HT29 and PCC)	Beclin 1/ATG7/ATG5-12/LC3-II	3-MA CQ	ATG3^B^ ATG5^B^	↑apoptosis ↑caspase 3	(129)
TPPOH-X SNPs	Lyso	Human colon cancer (HT29)	LC3-II increase	3-MA		↑apoptosis ↑caspase 3 ↓LC3-II	(164)
Verteporfin	Mito	Murine hepatoma cells (1c1c7)	PI3K/AKT/mTOR	CQ	ATG7^B^	↑apoptosis	(165)

Lyso, lysosome; memb, intracellular membranes; mito, mitochondria; ER, endoplasmic reticulum; 3-MA and wortmannin, Class III PI3K inhibitors; Bay11, NFκB inhibitor; CQ and BAF-A1, inhibitors of lysosome function; ^A^: siRNA; ^B^:shRNA; ^C^: CRISPR/Cas9.

**Table 2:** PDT-mediated autophagy: death routine and modulatory guidelines.

PS	Target	Experimental model	Molecular mechanism	Autophagy flux and death routine	Autophagy modulation	Outcome	Ref.
5-ALA	Mito	Human lung cancer (CL10 and PC12)	AMPK/MAPK/mTOR	Boosting: AMCD?	3-MA	↑survival ↓caspase-9 and 3	(104)
Verteporfin	Mito	Human prostate cancer (PC3)	Inhibition of autophagosome formation	Dysfunctional: AACD	BEZ235 LY-294002	↑apoptosis ↓cleaved PARP1 ↑LC3-II	(144) (145)
Ce6	Mito	Human breast cancer (MCF7 and MCF7/ADR)	?	?	3-MA BAF-A1	↑survival	(166)
		Human breast cancer (MDA-MB-231 and MCF7)	AMPK	Boosting: AACD	3-MA + 2-DG	↑apoptosis ↑pAMPK ↓Beclin 1 ↓LC3-II ↓Tumor mass	(167)
DMMB	Mito Lyso	Human cervix carcinoma (HeLa), hepatocarcinoma (HepG2), and melanoma (SKMEL18 and 25)	Mitophagy/Lysosomal dysfunction	Dysfunctional: AACD ↑PRKN, ↑SQSTM1, ↑LC3II, ↑autolysosomes accumulation	3-MA BAF-A1	↓survival Non-changed	(59)
Hypericin high dose (500 nM)	ER	Human oxaliplatin-resistant colon cancer (HCT116, HCT8) and HCT116/L-OHP murine model	GRP78/CHOP/AKT	Boosting: AACD ↓SQSTM1, ↑LC3II/I	3-MA ATG5* Beclin 1*	↑out-growth ↑Ki67 ↓oxaliplatin sensitization	(128)
MPPa	ER	Human osteosarcoma (MG-63)	JNK pathway	Boosting: AMCD? ↓BCL-2, ↑ Beclin 1, ↑LC3II/I, ↓SQSTM1	3-MA CQ	↓apoptosis ↓LC3-II ↓caspase 3	(168)
		Human breast cancer MDA- MB-23 and murine model	PERK/eIF2α/CHOP	Boosting: AACD ↑ Beclin 1, ↑LC3BII/I, ↓SQSTM1	3-MA	↓apoptosis ↑out-growth ↓LC3B-II	(62)
		Human osteosarcoma (MG-63)	PERK/IRE1α/CHOP CHOP/AKT/mTOR	Boosting: AMCD? ↑LC3II/I,↓SQSTM1	Rapamycin	↑apoptosis ↓pMTOR ↑LC3-II ↓SQSTM1 ↑caspase 3 ↑ cleaved PARP1	(147)
mTHPC Foscan®	ER	Human breast (MCF7), lung (A-427), oral cavity (BHY), esophagus (KYSE-70), bladder (RT-4), and cervix (SISO) cancer	GRP78/LC3-II	Boosting: AMCD? ↑LC3II/I	Wortmannin PSA E-64d	↓apoptosis ↓LC3-II ↓caspase-9 ↓caspase-3 ↑LC3-II	(56) (155)
NPe6	Lyso	Murine hepatoma (1c1c7)	Lysosomal dysfunction	Dysfunctional: AACD? ↑LC3II/I, ↑vacuolization	ATG7*	↑survival ↓caspase activation	(169)
N‐TiO_2_ nanoparticles	Lyso	Human melanoma (A375)	Impairment of lysosomal fusion with autophagosome	Dysfunctional: AACD ↑LC3II/I, ↑SQSTM1 ↑RIPK1, ↑HMGB1	3-MA BAF-A1 Necrostatin-1	↓necroptosis ↑cellular rescue (90%)	(170)
TPPS2a	Lyso	Human cervix carcinoma (HeLa)	Lysosomal dysfunction	Dysfunctional: AACD ↑LC3II/I, ↑vacuolization	3-MA	Slightly decrease	(35)
TPGS/dc-IR825	Mito	Human lung cancer (A549) and xenografts	Mitophagy/AMPK	Boosting: AACD ↑PINK1, ↑PRKN, ↑LC3II/I, ↑SQSTM1, ↓ATP	CQ 3-MA	↑survival ↓PINK1 ↓pAMPK	(141)
WST11 TOOKAD®	Lyso	Murine hepatoma (1c1c7)	Lysosomal dysfunction	Dysfunctional: AACD? ↑LC3II/I, ↑vacuolization	ATG5* ATG7*	↑survival ↓caspase activation	(169)

Lyso, lysosome; mito, mitochondria; ER, endoplasmic reticulum; 3-MA, LY-294002, and wortmannin, Class I/III PI3K inhibitors; BEZ235, Class I PI3K/mTOR inhibitor; rapamycin, mTORC1 inhibitor; BAF-A1, CQ, E-64e, PES, inhibitors of lysosome function; * genetic silencing/knockout.

### The Pro-Survival Autophagy Role

In general, the therapeutic effects of PDT are related to apoptosis or necrosis, and autophagy might be a double-edged sword, depending on the type of photosensitizers and cells (19). Instead of promoting cell death *per si*, autophagy often accompanies the cellular demise by PDT, as a last attempt of cells to cope with oxidative stress and to survive. Several *in vitro* and *in vivo* reports have demonstrated considerable evidence that autophagy plays a pivotal cytoprotective role that virtually occurs along with other RCD, like apoptosis, necroptosis, necrosis, or parthanatos (54, 55, 60, 74, 100–103, 128, 129, 146, 161–165, 171–174). The description of such cell death mechanisms will not be considered herein.

Autophagy is a key point on survival and tumor adaptation, whose inhibition decreases anti-apoptotic proteins’ expression (e.g. BCL-2 and survivin) or increases pro-apoptotic proteins, such as BAX, leading to tumor sensitization to photo-stress, e.g. 5-ALA-PDT (68). However, this protective response may be compromised *via* photooxidative-mediated NFκB activation through induction of an adaptative AKT/mTOR/S6K response that leads to the alleviation of necrotic cell death (103). The apoptotic machinery (e.g. upregulation of cytochrome *c* release, BAX, caspase-3, and PARP1) was found to occur accompanying the protective autophagic signals in response to Photosan II-PDT *via* activation of the AMPK pathway or suppression of the AKT/mTOR signaling (102). Notably, the protective autophagy is responsible for cell adaptation and delay of PARP1-mediated apoptosis at low dose hematoporphyrin-PDT (101).

Autophagy was found to protect photosensitized cells from oxidative damage triggered by several photosensitizers, like 5-ALA (100, 103), chlorophyllin e4 (55), chlorophyllin-f (54), hypericin (60, 128), hypocrellin A (146), Pc13 (74), Photofrin™ (161, 162), protoporphyrin IX (129), and porphyrin IX (163), TPPOH-X SNPs (164), and verteporfin (165). This cytoprotective autophagy can be alleviated trough chemical (e.g. BAF-A1, CQ, 3-MA, or wortmannin) and genetic inhibition of essential autophagy-related genes (e.g. *ATG3*, *ATG5*, *ATG7*, or *Beclin 1*) or autophagy regulators (e.g. *CHOP*), leading to significant suppression of PDT-resistance of tumor cells (**Table 1**).

Thereby, photoinduced cellular stress could be targeted to further death through negative modulation of autophagy. Xiong et al. demonstrated a promisor therapeutic association-targeting autophagy to overcome PDT-resistance of colon cancer xenografts (102). Protoporphyrin IX-PDT in colorectal cancer stem-like cells (CCSCs) failed to initiate out-growth in almost 70% when associated with autophagy inhibitors (e.g. CQ and *ATG5* shRNA), compared to 25% in PDT alone. Thus, autophagy inhibition can be considered as a target to deal with adaptation or resistance to photooxidative stress, leading to higher antitumorigenicity of PDT in tumor-xenografts (102, 129).

Whereas most of the PDT-protocols trigger autophagy as cytoprotective, fewer propose to activate autophagy as a death routine in cells succumbing to photooxidative stress, as summarized in **Table 1**. By analyzing reliable experimental studies, we propose in the next section to cope with the challenge to distinct autophagy-associated death modes, considering the high variability of cellular responses and different types of PDT protocols.

### The Pro-Death Autophagy Role

In general, photoinduced cell death in the mammalian cells is preceded or accompanied by autophagic vacuolization, a morphological alteration that may be considered as an example of the widespread belief of a “*type II programmed cell death*” or “*autophagic cell death*” (175). However, both terms are unappropriated following recent guidelines (160). On July 15, 2020, a Medline search of “*autophagic cell death*” or “*autophagic death*” and “*PDT*” or “*Photodynamic Therapy*” yielded 17 entries, which constitutes a fraction - close to 10% - of all 184 articles published on the topic “*autophagy*” in the PDT field. This led us to reflect on the expression “*autophagic cell death*” (57) after photodamage. Autophagy can protect cells and help them to tolerate the photodamage (60, 129); however, if there is a high level of autophagy or blockade flux, “*autophagic cell death*” could probably occur. Herein, we review this paradigm and – polemically – raise doubts about the existence of “*autophagic cell death*” mediated by PDT.

Incontestably, converting a protective autophagic mechanism to a destructive or lethal avenue is now being well-defined as autophagy-dependent cell death (ADCD) as postulated by the international committee on cell death (176). Using ADCD term one would postulate that the photoinduced death is autophagy-regulated through its machinery and thereof components, whereas its pharmacologic or genetic lessening would lead to less death regardless of other RCD mechanisms. Even though most of the reports have evaluated increased autophagic flux and puncta vacuoles, none of them formally establishes autophagy itself (or ADCD) as responsible for photo-induced cell death. Consequently, before ascribing a direct death role to autophagy, it is recommended to determine the machinery efficiency status by generally inhibiting the autophagy pathway using genetic approaches (knockdown or knockout based in siRNA, shRNA, or CRISPR/Cas9), see **Table 2**.

Some reports have shown that depending on the stress level, the autophagic apparatus might intrinsically contribute to other cell death programs, like apoptosis or necroptosis (35, 56, 59, 62, 104, 128, 141, 144, 145, 147, 155, 166–170), see **Table 2**. In spite of not inducing cell death *per si*, the RCD routine autophagy-mediated cell death (AMCD) may be significantly rescued by chemical autophagic inhibition (e.g. CQ, 3-MA, or BAF-A1) and/or genetic manipulation (e.g. ATG7, Beclin 1), as reported for some drugs (e.g. QW24, PTC-209, or sodium butyrate) (177–179).

During photooxidative damage autophagic machinery seems to play a key role regarding the increase in death mediators, probably leading to AMCD together with apoptosis (56, 147, 168). Another RCD routine linked to autophagy has been proposed, i.e., autophagy-associated cell death (AACD, which may or not occur alongside other cell death modalities, like apoptosis (**Table 2**). AACD commonly relates to the impairment of the early (144, 145) or the late stages of the autophagy flux (35, 59, 169, 170). Based on recent evidence, we propose that the terms “*autophagic cell death*” or “*autophagy death*” should be substituted to “AMCD” or “AACD”. However, one should initially consider the main differences between AMCD and AACD in tumor cells succumbing to photooxidative stress. Unlike AACD (35, 59, 62, 128, 141, 167, 170), AMCD may require the autophagic machinery to intrinsically regulate apoptosis (56, 104, 147, 155, 168), as summarized in **Table 2**.

The engagement of autophagy as a death route does not occur naturally, instead, quite specific experimental conditions should be followed, including the PDT exposure dose, type of protocol sub-sequentially or parallel photodamage on intracellular targets), type of targeted organelle (e.g. lysosomes or ER), availability of cellular machinery to evade apoptosis. Whereas in the case of cytoprotective autophagy the pharmacologic or genetic autophagic lessening sensitizes tumor to higher PDT-photoinduced death (**Table 1**), in the case of pro-death autophagy may occur either no effect or substantial alleviation of the PDT-photokilling (**Table 2**). Nevertheless, autophagy as a death routine remains allusive and still requires more studies.

The autophagy-related death can be either related to AMCD or AACD, depending on the dose, physicochemical properties, and the intracellular specificity. Lange et al. showed different autophagy responses concerning the Foscan®-PDT doses (e.g. LD_50_ versus LD_90_) (155). While LD_50_ dose leads to moderate ER-stress with less apoptotic cell death and probable autophagic response, high-dose PDT (i.e., LD_90_) by damaging proteins involved in the autophagic machinery triggers pro-death autophagy associated with activation of apoptotic hallmarks, such as cleaved PARP1, phosphatidylserine membrane externalization (155). A similar regulation was described in mutated caspase-3 breast cancer cells (e.g. MCF7) (56). While autophagosome formation accompanies cleavage of pro-caspase 7 and PARP1 at the LD_90_ dose´s Foscan®-PDT, chemical inhibition of the autophagy flux lessen the pro-death autophagy, leading to a decrease in procaspase activation and less cytotoxicity (56). The MPPa-PDT also may activate pro-death autophagy *via* a ROS-dependent JNK/Beclin 1 pathway, which intrinsically enhances procaspase-3 activation (168). By suppressing the early (e.g. 3-MA) or late stages of the autophagic process (e.g. CQ), tumor recurrence may increase by up to 70% (168).

Recent reports revealed that MPPa- ER photo-stress could intrinsically regulate the pro-death autophagy *via* a PERK/CHOP/AKT/mTOR signaling, with consequent boosting autophagy flux and activation of PARP1, procaspase 3 and 12 (62, 147). Probably, as a secondary response of mTOR on the phosphorylation of S6K or 4EBP1, the ROS-mediated effect on the PERK pathway leads also to cell arrest, decrease in invasion and migration, due to respectively, downregulation on cyclins (A, E and B1) and metalloproteinases (MMP-2 and -9) (147). Such MPPa-triggered downregulation of MMP-2 and -9 was due to the ROS-mediated inhibition of AKT/NFκB/mTOR signaling with suppression of the metastatic behavior of breast MCF-7 cancer cells in xenografts (180). Chen et al. demonstrated that rapamycin (mTOR inhibitor) enhances the phototoxicity related to MPPa-mediated pro-death autophagy with a consequent decrease in SQSTM1/P62 levels and increased cleavage of procaspase-3 and PARP1 (147). Even though this pro-death autophagy role should be more investigated regarding the elicitation of the autophagic machinery to cleavage PARP1 and procaspases, these findings are suggestive of an AMCD routine.

Lin et al. revealed that the correlation between AMCD and ER photo-stress occurs *via* CHOP following hypericin-PDT at high doses (128). They proposed that pro-death autophagy occurs in the case of high ER photo-stress, which may relieve chemo-resistance towards oxaliplatin (128). Paradoxically, a low level of ER photo-stress mediated by hypericin-PDT leads to the opposite role of the autophagy process (i.e., pro-survival), *via* downregulation of AKT/mTOR signaling in HeLa tumor cells, which enhances PDT-mediated death by 50% after negative modulation of autophagy (e.g. *ATG5* siRNA or 3-MA) (60). Thereby, depending on the level of damage (low or high dose) mediated by hypericin-PDT, autophagy may contribute distinctively in apoptosis-resistant tumor cells (128).

The type of activation mechanism (e.g. AMPK) regardless of the type of PS (i.e., 5-ALA or Ce6) also leads to pro-death autophagy (104, 167). Although this interpretation is correct, it is still not possible to correlate pro-death autophagy with PS type and subcellular localization. PDT activates the pro-apoptotic MAPK/JNK/p38α pathway (100, 114) but also negatively regulates the AMPK phosphorylation (104). Parallel to AMPK activation, there is a decrease in the caspase-3 activity, an increase in the ATP depletion, and deactivation of the MAPK1/3 pathway (104). That MAPK deactivation would lead to mTOR activation, with consequent inhibition of autophagy, is contrary to the sustained AMPK activation that maintains elevated as an adaptative response to 5-ALA-PDT (104). Consequently, AMPK seems to be a key point in the PDT-response and autophagy activation. Indeed, through AMPK activity abrogation by compound C or VPS34 inhibition (3-MA mediated) the cell survival might be partially rescued, possibly due to the autophagy-independent mitochondrial photodamage (104). Corroborating this finding, Ce6-PDT also activates AMPK that is further enhanced upon glycolysis inhibition by 2-DG, with consequent tumor regression *in vitro* and *in vivo* (167). The AMPK hyperactivation relates to high ATP depletion, which leads to an increase in Beclin 1 and LC3 lipidation that could be lessened by 3-MA (167). Especially in caspase-3 mutated cancer cells resistant to either multidrug (e.g. doxorubicin, cisplatin, and paclitaxel) or Ce6-PDT when the autophagic flux is chemically inhibited (e.g. 3-MA or BAF-A1), the tumor relapse increases up to 50% (166). It seems that the hyperactivation of the AMPK pathway relates to boosted autophagy triggered by photooxidative damage on mitochondria (**Figure 3**). However, it is important to emphasize that the activation mechanisms of AACD remain elusive and poorly understood.

Overall, excessive, or impaired mitophagy/autophagy appears to trigger the AACD routine. The boosted mitophagy activation mediated by TPGS/dc-IR825-PDT exceeds the degradative function of autolysosomes, ending up with huge vacuolization and degradation impairment, as well as depletion of ATP, activation of AMPK pathway, and bioenergetic catastrophe (141). Such effects were also observed in xenografts models when TPGS/dc-IR825 nanomicelles were intravenously injected into tumor-bearing mice showing high tumor accumulation and retention (141). TPGS/dc-IR825 nanomicelles showed a remarkable *in vivo* therapeutic efficiency leading to total tumor remission, possibly related to their minimized cellular-extrusion (141).

**Figure 5:**
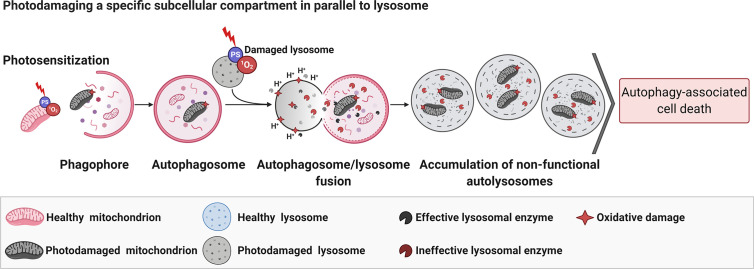
Parallel photodamage in mitochondria and lysosome evolves to autophagy-associated cell death. The PDT-mediated photodamage in mitochondria and lysosomes *per si* leads to efficient autophagy-associated cell death regardless of the chemical modulation of autophagy flux (i.e., BAF-A1 or 3-MA) (59). Figure created with BioRender.com.

When photodamage reduces the number of functional lysosomes and promotes their total disruption (59), or avoids their fusion with autophagosomes (170), AACD is activated. If the lysosomes are slightly photodamaged, only enough to enable an autophagic pro-survival response (probably due to lysophagy), there is a restoration of homeostasis (59). The pro-survival autophagy triggered by PDT (PS at low doses) can be promptly switched to AACD when parallel mitochondrial membrane damage occurs (59). In line with this notion, lysosomes have been considered as promisor targeted-organelle to PDT, even much more when parallel damage in the mitochondria membrane is mediated (59). Some reports corroborated with this premise (181, 182). Following this concept, the PDT-triggered mitophagy activation would fail in the context of lysosomal impairment, which evolves to AACD (**Figure 5**) (59).

Another path to trigger AACD was recently reported (170). Whereas N‐TiO_2_-PDT induces efficient autophagic flux in the dark condition, its photo‐activation compromises pro-survival autophagy. The replacement of the cytoprotective response mediated by photooxidative stress relates to the impairment of the lysosomal fusion with autophagosomes (170). Consequently, there was an increase in ROS production with consequent elicitation of RIPK1/HMGB1‐related necroptosis, which is abrogated upon treatment with necrostatin-1, a specific inhibitor (170).

The PDT-triggered molecular mechanisms differ concerning the pro-death autophagy routine, i.e., AMCD or AACD, and the mechanistic framework must be carefully considered before choosing the type of autophagy modulation (e.g. activation or inhibition). For instance, even targeting the same organelle (i.e., lysosomes), the AACD elicitation might differ upon the 3-MA inhibition in the early autophagy. Whereas the photoinduced lysosomal dysfunction promoted by DMMB evokes *per si* tumor death regardless of 3-MA (59), the impaired lysosome/autophagosome fusion mediated by N‐TiO_2_-PDT leads to a high level of tumor relapse (90%) (59). Thereby, to efficiently relieve tumor recurrence, a better choice should be the lysosomal inhibitor BAF-A1, which slightly increases the N‐TiO_2_ phototoxicity (170). Meanwhile, when PDT increases AMCD, the boosting autophagy triggered through mTOR inhibition (e.g. rapamycin) should be the best direction to deal with MPPa-PDT-resistance (147). Moreover, the secondary effects regarding mTOR suppression would lessen any invasive or migratory activity of tumor cells (147).

Although autophagy plays a protective role in murine tumor cells photosensitized with lower concentrations of verteporfin (165), at higher concentrations it switches pro-survival autophagy to AACD probably by the inhibition of autophagosome formation in human prostate cancer cells (144). Thereby, in high-phototoxicity doses that compromise autophagy flux instead of inhibiting autophagy it is preferable to modulate its activation through treatment with dual Class I PI3K/mTOR inhibitor, e.g. BEZ235 (144), or pan-Class I PI3K inhibitor, e.g. LY-294002 (145). BEZ235 markedly increased growth inhibition of PI3K mutated-cancer cells (183). These findings highlight that verteporfin-PDT is an independent cancer treatment strategy, capable of overcoming pro-autophagy to deal with cancer resistance (e.g. against chemotherapy and radiation). However, further *in vivo* studies are still urgent to determine whether such a combination will lessen tumor out-growth.

Based on these pieces of evidence, we can conclude that the efficiency and rate of engagement in causing death after PDT depend on the cell type, the photosensitizer type, protocol details (concentration, light dose, targeted-organelle, and others). As revealed by pre-clinical studies, both AACD and AMCD can be chemically or genetically modulated to increase PDT outcomes, and therefore both mechanisms should be considered as promisor ways to deal with clinical tumor recurrence.

## The Improvement in the Clinical Outcome of Cancer Patients Mediated by PDT

Aside from the PDT-mediated photodamage that intrinsically correlates with regulated cell death, PDT also plays antitumor immunological activity, involving activation of CD^+4^ and CD^+8^ helper T lymphocytes, endothelial damage, the release of inflammatory mediators and cytokines (184–188). Thereby, along with the engagement of cell death PDT outcomes, tumoral remission is also due to its modulatory role in the immune response (187), which could control the disease´s progression to distant sites. Several preclinical pieces of evidence pointed out the promising role of PDT as a therapeutic strategy for tumor local or distant remission supporting; the oncology community also moved forward in the clinical field. Consequently, several clinical trials have been conducted or are in progress. According to *Clinical Trials.Gov*, 186 intervention studies have been carried out so far (189).

Despite all favorable oncological applications of PDT, it still raises urgent debate in medical practice. We shall, therefore, summarize the key issues concerning clinical outcomes, tolerability, and efficacy of PDT using e.g. 5-ALA, HAL, Photofrin™, Foscan®. We have considered only completed or terminated clinical trials, which enrolled at least three patients, most of them had the involvement of apoptosis or necrosis, and we will argue how the regulation of autophagy could improve clinical outcomes.

Basal cell carcinoma (BCC) continues to have increased incidence rates worldwide, especially in Australia where there has been a 4.4-fold increase (190). According to a network meta-analysis of non-melanoma skin cancer treatment, the surgical excision has been considered as the optimal approach with high efficacy, considering the complete response and complete lesion clearance, with moderate adverse effects (191). However, the risk of developing a subsequent lesion in three years after the first one is elevated ranges from 33% to 70%, which probably evolves from compromised histological margins (192). Indeed, even after early surgery intervention, tumor recurrence was observed in 50% of the BCC patients (~7 months after first intervention) (193). In addition to the poor or unacceptable long-term cosmetic outcomes, wide surgical excisions might sometimes require surgical reconstruction (194).

Contemplating the preserved cosmetic effect and safety for treating non-melanoma skin cancer, PDT is highlighted as an alternative strategy to treat BCC (195, 196). According to the randomized phase 3 trial (NCT02144077), the PDT protocol employing the 5-ALA formulated through a non-emulsion gel BF-200, promoted an effective remission response (93.4%) in BCC lesions, with a low cancer recurrence of 8.4%, after a one-year follow-up (197). The trial showed side effects with mild to moderate intensity regarding tolerability and safety, including pain at the treatment site. Another clinical trial corroborates the favorable outcome of PDT for BCC treatment. In a non-randomized phase 1 trial (NCT00985829) with the enrolment of 28 participants, 5-ALA-PDT lead to complete (32%) or partial (50%) remission, without considerable cosmetic impairment, having only low cases of local pain (7.1%). Even though 5-ALA-PDT is a promising therapeutic avenue to tackle BCC, some details of the protocol were not considered or even described, including exposure time and thickness of the photosensitizer applied to the skin, the specific wavelength of the light used, and the clinical outcome of BCC concerning its histologic subtype, e.g. pigmentary, superficial or nodular. Therefore, it is difficult to analyze the reasons for partial remission response (<50%) (NCT00985829). Proper establishment/definition of the dosimetry parameters could improve this result (197). Considering tumor-adaptative response related to sustained AMPK signaling (104), drug-efflux (89) or iNOS/NO axis (123), the combination of 5-ALA-PDT with positive modulators of the autophagic machinery (e.g. rapamycin), regulators of iNOS and drug-extrusion, NO scavengers, or NFκB inhibitors should be considered to increase clinical outcomes (93, 100, 103, 114–120, 147).

In general, the therapeutic approach to tackle head and neck tumors is difficult, considering that recurrence or even the remaining disease may occur in over 40% of the treated patients (198). This scenery maybe even more dramatic in severe cases, including those related to surgeries following neo-adjuvant treatment, or even, in the case of adjuvant radiotherapy. To overcome such difficulties related to internal tumors, the PDT protocol was improved based on the facility to percutaneously deliver light using multiple laser fibers, which are inserted directly into head and neck tumors, named Interstitial photodynamic therapy (iPDT). A phase 1-2 study attempted to assess the efficacy of iPDT using Foscan® as a photosensitizer. This strategy was considered as an alternative rescue therapy to treat recurrent head and neck tumors before surgery, radiotherapy, or chemotherapy (199). After 1 month of follow-up, 20% of the 45 patients treated obtained a complete response (e.g. free disease), whereas half (53%) experienced symptomatic relief (bleeding, pain, or decreased tumor volume). Among those patients with complete response, 33% died due to recurrence disease within an interval of 17 to 32 months. Meanwhile, 56% survived during follow-up time (10-60 months). Notably, 73% of patients survived for at least 16 months. Adverse events such as pain and edema for 2-4 weeks was reported (199). To improve clinical outcomes, the combination of Foscan®-PDT with late inhibitors of autophagy flux might be considered in future studies, which beyond increases to AACD may improve antitumor immunological activity. This combined approach may increase the proteotoxicity and calreticulin surface exposure, instigating a series of immune responses including DC maturation, CD^8+^ T cell proliferation, and cytotoxic cytokine secretion (112, 125, 155, 200).

In head and neck tumors, several reports are using PDT as a treatment option, from early-stage tumors to those without other therapeutic alternatives. Complete remission rates range from 68% to 95% (201). According to the open-label phase 2 trial (NCT00453336), the PDT protocol employing the Photofrin™ evolved a better clinical outcome regarding the lesion location, the histopathological and clinical staging, thereby, less aggressive/invasive lesions located in the oral cavity at early stages of the disease. Briefly, the casuistic comprised 45 patients showing lesions in the oral cavity (53.3%), larynx (40%), or other lesions (6.7%). Concerning the histological subtypes, 22/45 related to squamous carcinoma, 13/45 to severe dysplasia, 9/45 *in situ* carcinomas, and 1/45 verrucous cancer. Upon a six month-based follow up, Photofrin™-PDT evolved completed cancer remission of 73% for less aggressive or invasive disease (e.g. severe dysplasia and *in situ* carcinoma), whereas it was of 50% for squamous carcinoma. On the other hand, clinical-stage squamous carcinoma (stage I) revealed 70% of complete responses, while more advanced stages obtained lower complete responses (i.e., 38%). This study counted to 45 adverse events, such as pain inside the oral cavity (53%) or moderate skin irritation (18%). Remarkably, after seven years of follow-up, 71% of patients obtained a desirable outcome; meanwhile, fewer patients required endoscopic resection (13%). Despite the difficulty of adequately managing the light device to provide proper dosimetry assessment, by following standard guidelines, it is possible to successfully tackle carcinomas at early stages, which after Photofrin™-PDT treatment, show cure rates in the oral cavity and larynx as high as 94% and 91%, respectively (202). Based on preclinical findings the tumor-resistance should decrease following Photofrin™-PDT combined with negative regulators of autophagy (e.g. 3-MA or BAF-A1) (161, 162).

The nonrandomized prospective clinical trial (NCT00530088) proposed to determine the efficacy of Photofrin™-PDT in the treatment for dysplasia, *in situ* carcinoma, or stage I carcinoma in the oral cavity and larynx. After following the patients for a mean period of 15 months, a significant and complete lesion remission was observed in 92% of the patients, with recurrence in only 13% of the treated cases. Adverse effects associated with PDT were transient local edema, pain, and phototoxic reaction (203). These findings corroborate with the PDT´s premise as an alternative and efficient strategy to treat cancers of the oral cavity and larynx since the protocols are already better defined.

HAL-PDT was investigated to treat cervical intraepithelial neoplasia of 262 patients during a randomized phase 2 clinical trial (NCT01256424) (204). In this study, 118 were diagnosed with CIN1 (low-grade squamous intraepithelial lesion), 83 with NIC2 (high-grade squamous intraepithelial lesion), the others were not considered as eligible for the study (i.e., NIC 3 or regular exam). Among those eligible, the frequency for high-risk oncogenic HPV (e.g. HPV 16/18) was 46% (6/13) and 37% (7/19) for CIN2 and CIN1, respectively. Aside from the significant and sustained tumor remission of 95% for CIN2, PDT also leads to a remarkable reduction of HPV infection. For instance, PDT undergoes clearance of high-risk HPV in 83% of CIN2 patients (5/6) compared to the control group (33%). Despite the favorable PDT response, adverse effects were reported in 125 patients and included vaginal discharge, local discomfort, and mild bleeding (204). In a phase 2 study (NCT00708942) involving 83 women diagnosed with CIN1, it was observed that the HAL-PDT was able to offer a complete cytohistological and HPV viral clearance in 90% of the patients, in a 6-month follow-up.

Even with PDT’s mechanism inactivates the HPV virus is elusive; it seems to be related to the host’s immune response. Studies suggest that there is an influence of antitumor immunity after PDT. This premise is based on experimental findings demonstrating the activation of dendritic cells and tumor-specific T response upon PDT. Beyond this immunity activation, PDT also triggers systemic inflammation causing oxidative damage and cytokines release (187, 188).

Studies carried out on lung tumors have shown that PDT might reduce airway obstruction and improve respiratory function (205, 206). The literature has reported several clinical studies highlighting PDT as a promising strategy to treat early-stage, superficial lung cancer, through a robotic transthoracic needle, and navigation bronchoscopy (205). The first clinical trial was conducted in 1993, through a prospective phase 2 trial on PDT using Photofrin II in which 84.8% cases of squamous cell carcinoma, centrally located, evolved a complete response after initial PDT-treatment (207). This favorable outcome extended for a median of 14 months (range 2-32 months). Aside from a lower frequency of side effects (e.g. photosensitivity in 2% of cases), PDT led to fewer cases of local recurrence in 4/50 (8%) cases during the 16-month follow-up. The multicenter phase 2 trial applying NPe6-PDT revealed a considerable outcome in patients succumbing with early-stage, lung squamous carcinoma (208). Again, PDT leads to a complete response in 85% of lesions but now with incredibly low skin photosensitivity. Based on these favorable findings, PDT with Photofrin II or NPe6 was approved in Japan as a suitable treatment for early-stage lung cancer, centrally located (205).

The uncontrolled, non-randomized, open-label, prospective, multicenter, phase 1 clinical trial (NCT03344861), performed in 10 patients, evaluated the safety of the tissue response to hematoporphyrin-PDT in solid lung tumor, previous to surgery. On the 15th day after PDT, patients were submitted to standard surgery, following macro and microscopic cancer evaluations. Despite the occurrence of side effects in 40% of patients, including hemorrhagic shock, anemia, and skin photosensitivity, the performance status and presence of inflammation suggest Photofrin™-PDT as a preoperative possibility in solid lung tumors. The same group conducted a phase 1, interventionist study (NCT02916745), in 5 patients diagnosed with non-small cell lung cancer or with lung metastasis. The objective was to assess the safety and viability of Photofrin™-iPDT by bronchoscopy intervention. The tumor remission with antitumor immunity after 6 months of iPDT was complete (20%) or partial (60%). In general, this PDT-mediated antitumor-immunity has been associated with the activation of dendritic and T cells (188).

The critical role of autophagy in cell biology and its considerable therapeutic potential against cancer recently received the oncology community’s attention. Autophagy generally promotes resistance to photodynamic therapy-induced apoptosis or necrosis and may serve as a strategy to improve its efficacy (129, 161). In line with this notion, PDT’s autophagy modulation would represent a potential therapeutic target for human cancer (209, 210). We must be aware that the PDT-phototoxicity as well as the type of autophagy induction is dose-dependent, if cytoprotective or pro-death, as discussed earlier.

## Major Challenges and Perspectives

The trajectory of clinical PDT for cancer treatment is somewhat peculiar and not straightforward. Many new photosensitizers have been designed and tested, showing relatively important improvements compared to preceding ones. However, few of them were approved by the FDA and others are undergoing clinical trials (17). Despite the FDA approval, until now no PS presented a magic bullet or exhibited all characteristics of an ideal PS. Photofrin™, Foscan®, ALA and HAL are still the most used photosensitizers in PDT, despite the several disadvantages presented by them (17).

Thus, somehow a significant part of the knowledge acquired is not reaching clinical protocols routinely. It is essential to call attention to this fact and ask: how can we get through it? We consider that one of the bottlenecks for expanding the PDT application in a clinical routine because of the biological system’s complexity. Toward this end, it would be necessary to stress the interaction of the PDT response regarding other intrinsic stressors, including cell stemness capacity, metabolic condition, cross-talking with the microenvironment and stroma, microbiota, genome instability, inflammatory and immune responses, vasculogenic mimicry, hypoxia, and other biochemical anomalies.

Another pivotal point comprises the discrepancy or even lack of a consensual or gold-standard procedure for PDT clinical practice concerning light dose exposure regimen, PS concentration, and the type of light device used. Also, topical products containing PS are lacking information about the exact amount applied to the skin and the time to be activated. All these points make it difficult to establish standard protocols that can be replicated in other studies. Furthermore, the progression-free survival investigation of clinical cases is still missing. We should also look for the PDT’s ability to prolong the patient’s time of life, instead of PDT being indicated only for the curative aspect.

Considering the premise that autophagy represents a therapeutic target to improve oncology clinical outcomes, future efforts should be made to the development of drugs with increased pharmacologic specificity beyond those commonly used in the current approach. Among all efforts, we have elicited mainly those focused on the development of novel autophagy inhibitors, whose consolidation into therapeutic regimens should be considered as a new avenue for the PDT antitumor field.

## Concluding Remarks

In this review, we discussed the significant progress in the comprehension of autophagy modulation in cells succumbing to photooxidative damage. Preclinical reports pointed out that autophagy targeting can be a key regulatory routine to improve clinical outcomes in oncology practice. The repurposing drugs have been considered, including mTORC1 inhibitors (e.g. temsirolimus, everolimus, and rapamycin), chloroquine, and BAF-A1. Several efforts have been made to deal with tumor resistance. Chemical or photochemical inhibition of lysosomal function seems to bear a promising strategy since autophagy machinery plays a pivotal role in tumor vulnerability. Towards the increase in the death-autophagy related to lysosomal photodamage (e.g. verteporfin-PDT) or ER-stress (e.g. MPPa-PDT), the positive regulation of autophagy (e.g. BEZ235, LY-294002, or rapamycin) is highlighted as a promisor way to deal with tumor resistance. Notably, the modulation of parallel photodamage in lysosomes and mitochondria is a favorable route to trigger AACD thoroughly. Therefore, the PDT-mediated autophagy associated-cell death may be considered as a new therapeutic avenue, even though it needs to be further explored in clinical trials.

## Author Contributions

WM revised literature, organized and took the lead in writing the main manuscript with contribution from all authors. RB revised the literature and wrote mainly the section about the clinical outcome in cancer mediated by PDT. MNS contributed to the section about regulated cell death mechanisms PDT-induced in cancer cells and clinical outcome in cancer mediated by PDT. DG contributed to writing the section about tumor resistance. MS and TL contributed to describing the procedure and photophysical mechanism in PDT. RI and MB contributed to the overall review mainly about PDT concepts. TT also revised literature, wrote the PDT section, and contributed to the overall manuscript. WM and TT coordinated all the work. All authors contributed to the article and approved the submitted version.

## Funding

The authors thank to Fundação de Amparo à Pesquisa do Estado de São Paulo (FAPESP) for grant numbers 2013/07937-8, 2016/23071-9, 2016/07642-6, and 2018/22922-0. Authors also acknowledge Programa de Apoio à Pós-graduação (PROAP) from Federal University of Uberlândia for financial support and Coordenação de Aperfeiçoamento de Pessoal de Nível Superior (CAPES) – Finance Code 001.

## Conflict of Interest

The authors declare that the research was conducted in the absence of any commercial or financial relationships that could be construed as a potential conflict of interest.

## Abbreviations

1400W, N-[3- (aminomethyl)benzyl]acetamidine; 2-DG, 2-deoxy-D-glucose; 3-MA, 3-methyladenine; 5-ALA, 5-aminolevulinic acid; ABC, ATP binding cassette; ABCB1, ATP Binding Cassette Subfamily B Member 1 or P-Glycoprotein 1; ABCB6, ATP Binding Cassette Subfamily B Member 6 (Langereis Blood Group); ABCG2, ATP Binding Cassette Subfamily G Member 2 (Junior Blood Group) or BCRP; AKT, Ser and Thr kinase AKT, also known as protein kinase B; APAF-1, Apoptotic protease-activating factor-1; ATF4, Activating transcription factor 4; ATF6, Activating transcription factor 6; BAF-A1, Bafilomycin A1; BAK, Bcl-2 homologous antagonist killer; BAX, Bcl-2 -associated X protein; BLC-2, BCL2 apoptosis regulator; BCL-xL, BCL2 Like 1; BID, BH3 interacting-domain death agonist; BPD, Benzoporphyrin or verteporfin; Ce6, Chlorin e6; CHOP, C/EBP homologous protein or DNA damage inducible transcript 3 (DDIT3); CisDiMPyP, *meso-cis*-di(N-methyl-4-pyridyl)diphenyl porphyrin dichloride; cPTIO, 2-4-carboxyphenyl-4,4,5,5-tetramethylimidazoline-1-oxyl-3-oxide; CQ, Chloroquine; DCA, Dichloroacetate; dc-IR825, Near-infrared cyanine dye; DMMB, 1,9 dimethylmethylene blue; eIF2α, Eukaryotic translation initiation factor 2A; EGFR, Epidermal growth factor receptor; ER, Endoplasmic reticulum; ERK, Extracellular signal-regulated kinases; H_2_O_2,_ Hydrogen peroxide; HAL, Hexaminolevulinate or 5-Aminolevulinic acid hexyl ester; HPPH, 2-(1-Hexyloxyethyl)-2-devinyl pyropheophorbide-a; HSP27, Heat shock protein 27; HSP70, Heat Shock Protein Family A (Hsp70) Member 4; HSP90, Heat shock Protein 90 Alpha Family Class A Member 1; HSPA5, Heat Shock Protein Family A (Hsp70) Member 5; iNOS/NOS2, Inducible nitric oxide synthase; JNK, c-jun N-terminal kinases; LAMP2A, Lysosome-associated membrane protein type 2A; LED, Light-emitting diode; MAP2K1, Mitogen-activated protein kinase kinase 1; MAPK1/ERK2, Mitogen-activated protein kinase 1; MAPK3/ERK1, Mitogen-activated protein kinase 3; MPPa, Pyropheophorbide-a methyl ester; m-THPC, *meta*-tetrahydroxyphenylchlorin; mTOR, Mammalian target of rapamycin; mTORC1, mTOR complex 1; NPe6, Mono-L-aspartyl chlorin e6 or talaporfin sodium; N‐TiO_2_, Nitrogen‐doped titanium dioxide; ^•^OH, Hydroxyl radical; $\left(O_{2}^{-\cdot }\right)$, Superoxide ion; $1O_{2}$, Singlet oxygen; p38α, P38 mitogen-activated protein kinase or Mitogen-Activated Protein Kinase 14; PARP1, Poly(ADP-ribose) polymerase 1; Pc13, Zinc(II) phthalocyanine Pc13; PEPT1, Oligopeptide transporter peptide transporter 1; PFKFB3, 6-Phosphofructo-2-Kinase/Fructose-2,6-Biphosphatase 3; PpIX, Protoporphyrin IX; PS, Photosensitizer; PSA, Pepstatin A; RIPK3, Receptor Interacting Serine/Threonine Kinase 3; ROS, Reactive oxygen species; SLC15A1, Solute Carrier Family 15 Member 1 or PEPT1; TPGS, D-α-tocopheryl polyethylene glycol 1000 succinate; TPPOH, 5-(4-hydroxyphenyl)-10,15,20-triphenylporphyrin; TPPOH-X SNPs, Silica nanoparticles (SNPs) coated with xylan-TPPOH conjugate (TPPOH-X); TPPS, Tetraphenylporphinesulfonate; TPCS_2a_, Disulfonated tetraphenyl chlorine; TPPS_2a_, *meso*-Tetraphenylporphine disulphonic acid disodium salt (adjacent isomer), WST11, Palladium bacteriopheophorbide.; TRIB3, Tribbles-related protein 3; ULK1, Unc51-like autophagy activating kinase 1; VMP1, Vacuole membrane protein 1.
